# Enhancing the photoelectrochemical performance of TiO_2_ photoanode by employing carbon nanoparticles as electron reservoirs and photothermal materials

**DOI:** 10.3389/fchem.2024.1471340

**Published:** 2024-09-24

**Authors:** Jing Huang, Yijie Huang, Puwen Guo, Yinchang Li

**Affiliations:** ^1^ Hubei Key Laboratory of Pollutant Analysis and Reuse Technology, College of Chemistry and Chemical Engineering, Hubei Normal University, Huangshi, China; ^2^ International Collaboration Laboratory of 2D Materials for Optoelectronics Science and Technology of Ministry of Education, Shenzhen University, Shenzhen, China

**Keywords:** photoelectrochemical, water oxidation, photoanode, electron reservoirs, photothermal

## Abstract

Photoelectrochemical (PEC) water splitting is regarded as a potential technique for converting solar energy. However, the fast charge recombination and slow water oxidation kinetics significantly have hindered its practical application. It is found that an elevation in operation temperature can activate the charge transport in the photoanodes. Here, a strategy was performed that carbon nanoparticles were employed to TiO_2_ nanorods, acting as electron reservoirs as well as photothermal materials. More specifically, a record photocurrent density of 1.62 mA cm^−2^ at 1.23 V vs. RHE has been achieved, accompanied by a high charge separation efficiency of 96% and a long-term durability for 8 h. The detailed experimental results reveal that under NIR light irradiation, the synergistic effect between electron storage and temperature rise leads to accelerated charge transport in the bulk and water oxidation kinetics on the surface. This research offers a new perspective on how to boost the PEC performance of photoelectrodes.

## Introduction

The capacity of photoelectrochemical (PEC) water splitting to produce hydrogen and oxygen from solar energy with a high theoretical solar-to-hydrogen (STH) conversion efficiency has garnered much attention in recent years (Wang et al., 2019; Hu et al., 2020; Xiao et al., 2020). At present, PEC water splitting is thought to be among the most promising methods for producing hydrogen and assisting in the future resolution of the energy dilemma (Shi et al., 2018; Lee and Choi, 2017; Zeng et al., 2021; Zhang J. et al., 2021a). However, compared to the two-electron water reduction reaction at the photocathode, the rate of the four-electron water oxidation reaction at the photoanode is much lower (Liu et al., 2023; Lv et al., 2022). Consequently, the rate-determining phase that controls the PEC water splitting reaction rate is the sluggish water oxidation reaction at the photoanode (Song et al., 2022; Tang et al., 2021; Zhang Z. et al., 2022b). Among numerous photoanode semiconductor materials, titanium dioxide (TiO_2_) has attracted widespread attention from researchers due to its excellent chemical stability, low cost, non-toxicity, and suitable water oxidation valence band position (Guo et al., 2019; Liu et al., 2021; Arunachalam et al., 2023; Chen et al., 2024). However, the severe photogenerated electron-hole recombination as well as the slow oxygen evolution kinetics of TiO_2_ greatly limit its PEC performance (Shen et al., 2022; Li et al., 2021).

Accumulating a large number of photogenerated holes on the photoanode surface under light irradiation is essential to accelerate the water oxidation process. Therefore, effective charge separation during PEC water splitting is necessary. By modifying the photoanode with oxygen evolution cocatalysts (OECs), it is possible to promote the water oxidation activity of the PEC water splitting by encouraging the charge separation of photogenerated electron-hole pairs (Zhang S. et al., 2022; Yoon et al., 2016; Lu et al., 2022; Zhang X. et al., 2021). According to previous research, carbon materials have the characteristics of good stability, good conductivity, easy charge storage, making them a highly competitive electronic storage material in energy storage devices (Fang et al., 2022; Yan et al., 2021; Tang et al., 2022; Hu et al., 2021). What’s more, carbon materials are known as photothermal materials with high photothermal conversion efficiency, which can convert near-infrared (NIR) light into thermal energy (Tian et al., 2021; Weng et al., 2020). The temperature of photoelectrode can be raised under NIR light irradiation by introducing photothermal materials, eliminating the need for extra heating devices. It is worth noting that raising the operating temperature is a feasible strategy to concurrently boost exterior catalytic activity and internal charge transfer, which can enhance PEC performance for the composite photoanode (Huang et al., 2023; Jin et al., 2019; Zhou et al., 2020).

Herein, carbon nanoparticles (CNPs) were grown on a TiO_2_ photoanode, acting as electron reservoirs and a typical photothermal materials, to form the CNPs-TiO_2_ (C-TiO_2_) composite photoelectrode. In this condition, CNPs act as the electron reservoirs to promote charge separation as well as typical photothermal material to accelerate charge transfer and surface water oxidation kinetics. With the synergistic effect between electron storage and temperature elevation, the C-TiO_2_ photoanode yields a photocurrent density of 1.62 mA cm^−2^ at 1.23 V vs. RHE under NIR light irradiation, which is more than two folders higher than that of the pristine TiO_2_ photoanode. The PEC water splitting system is stable without obvious decline after 8 h of continuous operation. Based on thorough investigations, a possible mechanism for synergistically enhanced PEC water oxidation on C-TiO_2_ under NIR light irradiation was proposed.

## Materials and methods

### Materials

Tetrabutyl titanate (C_16_H_36_O_4_Ti), hydrochloric acid (HCl), glucose (C_6_H_12_O_6_), acetone (C_3_H_6_O), ethanol (C_2_H_6_O), and sodium hydroxide (NaOH), were provided by Sinopharm Chemical Reagent Co., Ltd. Fluorine-doped tin oxide (FTO) glass (2 mm × 15 mm × 20 mm) was purchased from Dalian HeptaChroma SolarTech Co., Ltd. FTO glass was cleaned with washed in acetone, ethanol and deionized water for 20 min each, consecutively in an ultrasonic bath before usage. Deionized water was used for the synthesis and rinsing of samples.

### Synthesis

#### Preparation of TiO_2_ photoanode

Using a hydrothermal method, pristine TiO_2_ NRs were produced on conductive FTO glass. First, a mixture of deionized water (12.5 mL), hydrochloric acid (12.5 mL), and tetrabutyl titanate (0.5 mL) was transferred into a Teflon-lined autoclave (50 mL) with a piece of cleaned FTO glass placed inside. After that, the autoclave was heated and kept at 150°C for 10 h. After the autoclave was cooled down to room temperature in air, the as-prepared TiO_2_ sample was taken out, deeply rinsed with deionized water, and dried in air. Then the sample was annealed at 450°C for 30 min (5°C/min).

#### Preparation of C-TiO_2_ photoanode

The C-TiO_2_ photoanode was prepared by a hydrothermal method. Briefly, 15 mM of glucose was transferred into a Teflon-lined autoclave with a TiO_2_ sample placed at an angle of around 60° inside. The autoclave was heated to 200°C and kept for 8 h. After the autoclave was cooled down to room temperature in air, the sample was taken out, washed with deionized water, and then dried in the oven at 60°C.

#### Characterizations

The morphologies of the samples were investigated by scanning electron microscopy (SEM, Hitachi, SU8010) with energy dispersive spectroscopy (EDS). The crystallinity and the phase compositions of the as-prepared photoanodes were detected by powder X-ray diffraction (XRD, Bruker AXS, D8 Focus) with Cu Kα radiation. The Raman spectra were recorded on all solid states with a laser source of 532 nm (Horiba Jobin Yvon HR800). The absorption behavior of the samples was recorded on an ultraviolet-visible spectrometer (UV-vis, Shimadzu, UV-3101PC) equipped with an integrating sphere attachment. The Fluoromax 4P spectrofluorometer (FS, Horiba, Fluoromax-4P) equipped with laser (λ = 380 nm) was used to detect the photoluminescence (PL) of the samples.

#### PEC characterizations

A standard three-electrode cell was used for photoelectrochemical tests carried out on the CHI 660E electrochemical workstation. The working electrode was the prepared photoanodes, with an actual working area of 0.25 cm^2^. The counter electrode was Pt foil, and the reference electrode was Ag/AgCl electrode. The 300 W Xe lamp was used as a simulated sunlight source (100 mW cm^−2^). Additionally, the electrolyte utilized was 1 M NaOH solution (pH = 13.8). The RHE potential is calculated through the following equation: E_RHE_ = E_Ag/AgCl_ + 0.0591 pH + E°_Ag/AgCl_, where E_RHE_ is the converted potential vs. RHE, E_Ag/AgCl_ = 0.1976 V at 25°C, and E°_Ag/AgCl_ is the measured potential vs. the Ag/AgCl reference electrode. The applied potential of linear sweep voltammetry (LSV) tests was from −1 V∼1 V vs. Ag/AgCl with a scanning rate of 10 mV s^−1^, and that of the stability test was 0.22 V vs. Ag/AgCl. Electrochemical impedance spectroscopy (EIS) was performed on an electrochemical workstation (CH Instruments Inc., CHI660E) with a frequency range from 0.01 Hz to 100 kHz with an amplitude of 5 mV. Mott–Schottky curves were also collected using an electrochemical workstation. A gas chromatograph (Shimadzu, GC-8A) was used to measure the amount of H_2_ and O_2_ every 30 min.

Recombination of charge carriers occurs in bulk and at the interface, resulting in two main losses of the photogenerated photocurrent (*J*_abs_). Therefore, the following expression represents the measured photocurrent during water oxidation: *J*_ph_ = *J*_abs_ × *η*_inj_ × *η*_sep_, where *η*_sep_ is the charge separation efficiency, and *η*_inj_ is charge injection efficiency at the photoanode surface, and *J*_abs_ is the photocurrent density corresponding to 100% internal quantum efficiency.

The photocurrent during sodium sulfite oxidation (*J*_sulfite_) was measured due to all holes can split to participate in the water oxidation upon reaching the electrode/electrolyte interface (*η*_inj_ = 1).

As a result, it is simple to determine the *η*_inj_ and *η*_sep_ using the following relationship:

$$\eta_{\text{inj}}=J_{\text{ph}}\;/\;J_{\text{sulfite}}$$

$$\eta_{\text{sep}}=J_{\text{sulfite}}\;/\;J_{\text{abs}}$$

## Results and discussion

**FIGURE 1:**
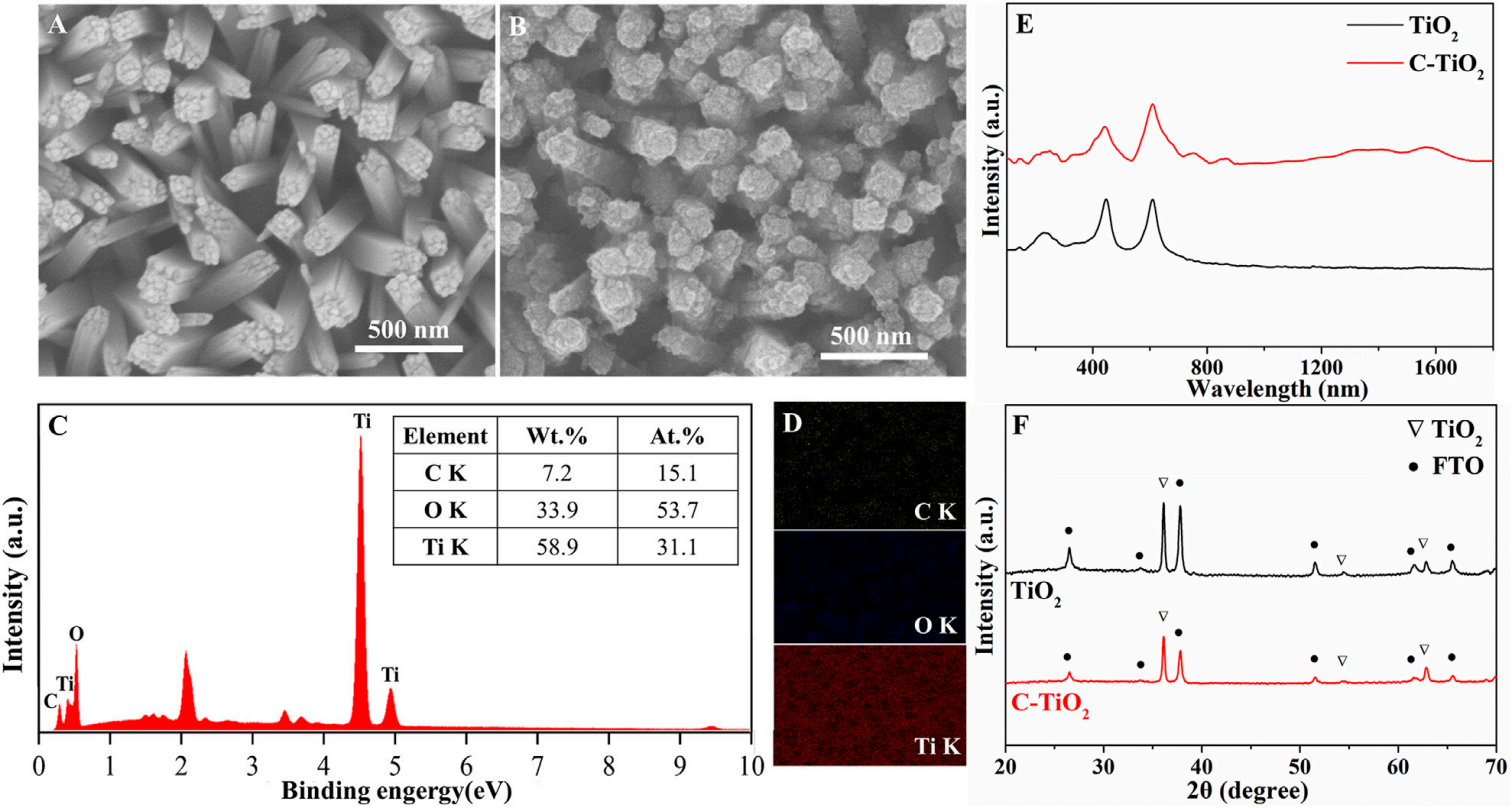
SEM images of the typical samples: **(A)** TiO_2_, **(B)** C-TiO_2_, EDS pattern **(C)** and the EDS elemental mapping **(D)** of as prepared C-TiO_2_ photoanode, Raman spectra **(E)** and XRD patterns **(F)** of the TiO_2_ and C-TiO_2_ photoanodes.

The morphology and elemental compositions of the synthesized C-TiO_2_ photoanode were studied with scanning electron microscope (SEM) and energy dispersive spectroscopy (EDS). Figures 1A, B exhibit the SEM images of TiO_2_ and C-TiO_2_. It is shown that carbon nanoparticles are uniformly coated on the surface of TiO_2_ nanorods. The elemental composition and content of C-TiO_2_ photoanodes were further investigated by EDS, As shown in Figures 1C, D, the weight percentage of elements present in the C-TiO_2_ photoanode are 58.1%, 38.9%, and 3.0% for Ti, O, and C, respectively. No other elements or impurities are found. The results clarify that the elements of Ti, O and C are present and uniformly distributed in the C-TiO_2_ photoanode. To further prove the existence of carbon in the as-prepared C-TiO_2_ photoanode, the Raman spectra of TiO_2_ and C-TiO_2_ (Figure 1E) are compared. In contrast to the spectrum of the TiO_2_ photoanode, there are two peaks at ∼1,576 cm^−1^ and ∼1,350 cm^−1^, which correspond to the characteristic peak of carbon materials (G band and D band), further confirming the existence of CNPs in the C-TiO_2_ photoanode (de Menezes et al., 2018). The X-ray diffraction (XRD) patterns of both TiO_2_ and C-TiO_2_ photoanodes (Figure 1F) are indexed to rutile TiO_2_ with no impurity peaks, except for the several peaks belonging to the FTO substrate. Thus, the introduction of CNPs does not cause structural change in the TiO_2_ nanorods. The absence of characteristic peaks of carbon XRD patterns is attributed to the poor crystallinity and the relatively low content in the C-TiO_2_ photoanode.

**FIGURE 2:**
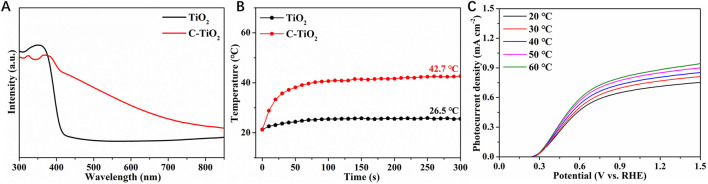
**(A)** UV-Vis diffuse reflectance spectra of TiO_2_ and C-TiO_2_ photoanodes. **(B)** The temperature-time curves of TiO_2_ and C-TiO_2_ measured in the electrolyte with NIR light. **(C)** The LSV curves of the TiO_2_ measured in the electrolyte at different temperatures.

The optical absorption properties of pristine TiO_2_ and C-TiO_2_ samples were investigated by UV-vis absorption spectroscopy. It is shown that the TiO_2_ exhibits an absorption edge at ∼420 nm (Figure 2A). After introducing CNPs onto TiO_2_ photoanode, the absorption edge exhibits a red shift, and the light absorption range has been extended to the near-infrared region. As one common type of photothermal materials, CNPs can effectively convert NIR light energy into local heat (Cui et al., 2023). Consequently, when exposed to NIR light, the local temperature of C-TiO_2_ photoanodes will be raised. Figure 2B displays the temperature evolution trends of TiO_2_ and C-TiO_2_ under 808 nm NIR light irradiation. The temperature increase of pristine TiO_2_ is modest and achieves a plateau at 26.5°C. The temperature of C-TiO_2_ reaches around 42.7°C, indicating that CNPs have an excellent photothermal conversion efficiency. Furthermore, Figure 2C illustrates the temperature dependence of PEC water oxidation for pristine TiO_2_ in 1 M NaOH electrolytes at various temperatures. Elevating the electrolyte temperature evidently results in a significant increase in current densities at 1.23 V vs. RHE, indicating that increasing the operation temperature may be a viable approach to boost TiO_2_ photoanodes.

**FIGURE 3:**
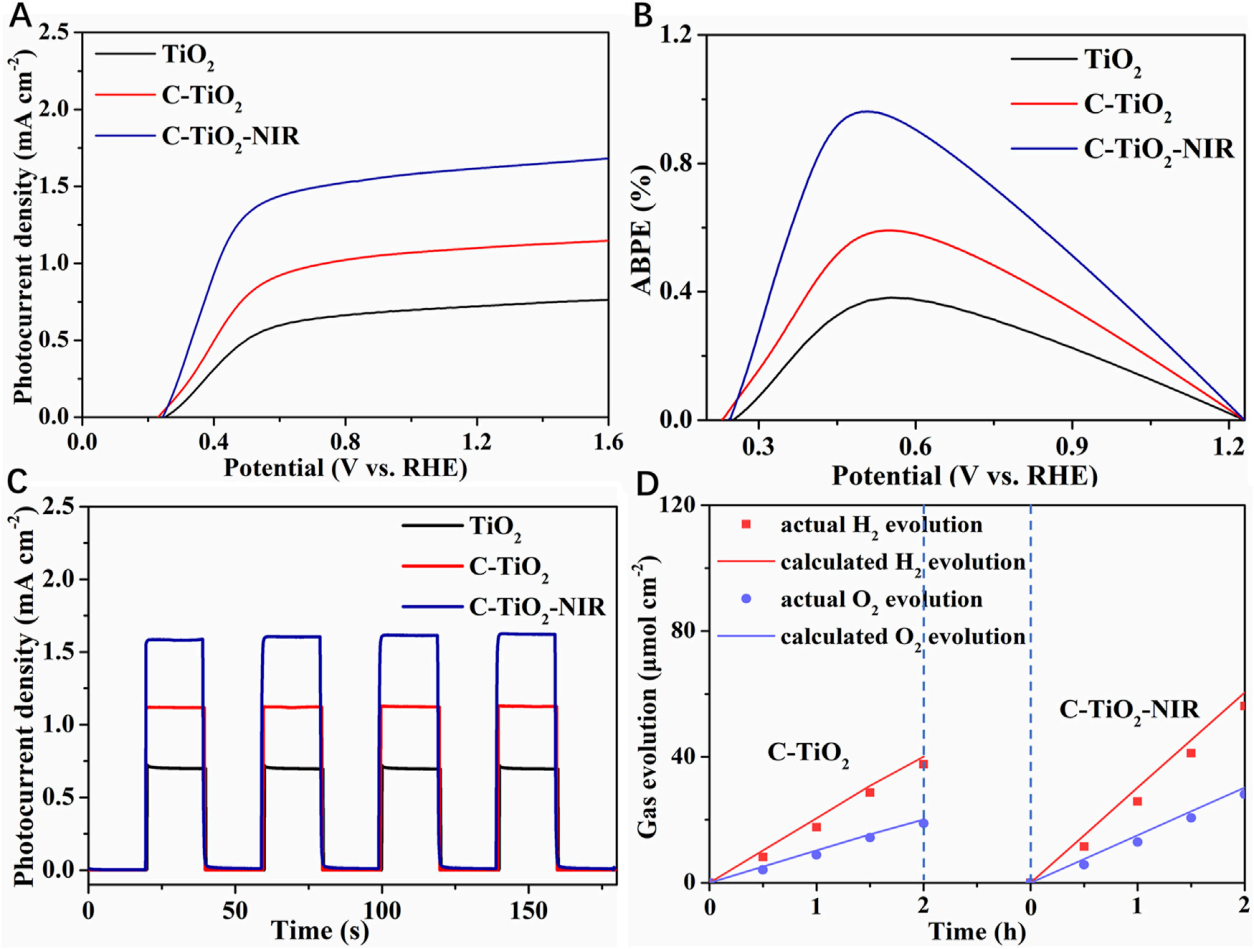
PEC performance of TiO_2_, C-TiO_2_ and C-TiO_2_-NIR: **(A)** LSV measurements. **(B)** ABPE curves. **(C)** Chopped linear sweep photocurrent-potential curves. **(D)** Evolution of H_2_ and O_2_ gases at an applied bias of 1.23 V vs. RHE.

In order to explore the PEC performance for TiO_2_ photoanode assisted by the photothermal effect of CNPs, linear sweep voltammetry (LSV) tests of TiO_2_ and C-TiO_2_ photoanodes with and without NIR light irradiation were conducted. As shown in Figure 3A, the photocurrent density of pristine TiO_2_ is 0.72 mA cm^−2^ at 1.23 V vs. RHE. After loading the CNPs cocatalyst on TiO_2_, the photocurrent density of the C-TiO_2_ photoanode reaches 1.10 mA cm^−2^ at 1.23 V vs. RHE, which is much higher than that of the pristine TiO_2_ due to the efficient charge separation between CNPs and TiO_2_. In particular, C-TiO_2_ obtained a more negative onset potential compared to pure TiO_2_. It is evident from the higher photocurrent density and negatively shifted onset potential that adding CNPs to the TiO_2_ photoanode is a workable method of improving its water oxidation ability. What’s more, when exposed to NIR light, the photocurrent density of C-TiO_2_ was further increased to 1.62 mA cm^−2^ at 1.23 V vs. RHE, confirming the positive effect of photothermal conversion of CNPs on the water oxidation process of the photoanode. Besides, the PEC performance of both C-TiO_2_ and C-TiO_2_-NIR photoanodes is significantly influenced by the loading amount of CNPs as shown in Supplementary Figure S1, which is modulated by a hydrothermal time of 6, 8, 10, and 12 h. It is evident that a decrease in photocurrent for C-TiO_2_ photoanodes results from the extended hydrothermal time of CNPs, which may be caused by the competition for visible light absorption of CNPs. And the interaction between TiO_2_ nanorods and the electrolyte solution is also reduced by the aggregation of CNPs.

According to the LSV results, the maximum applied bias photon to current efficiency (ABPE) for C-TiO_2_-NIR reaches up to 0.96% at 0.50 V vs. RHE (Figure 3B), while that is only 0.38% at 0.56 V vs. RHE for the pure TiO_2_ photoanode and 0.59% at 0.55 V vs. RHE for C-TiO_2_. Figure 3C shows the chopped photocurrent density-voltage curves of TiO_2_, C-TiO_2_ and C-TiO_2_-NIR. As expected, the photocurrent densities of TiO_2_ photoanode are improved after CNPs deposition. The enhanced photocurrent density for the C-TiO_2_ nanorods is attributed to the accelerated charge separation caused by CNPs acting as an electron storage layer. Notably, higher photocurrent for C-TiO_2_-NIR arises from improved temperature induced by CNPs acting as photothermal materials. Finally, photocurrent increases due to the enhanced photocarrier separation and transport in the bulk, and accelerated water oxidation on the surface, as discussed below.

**FIGURE 4:**
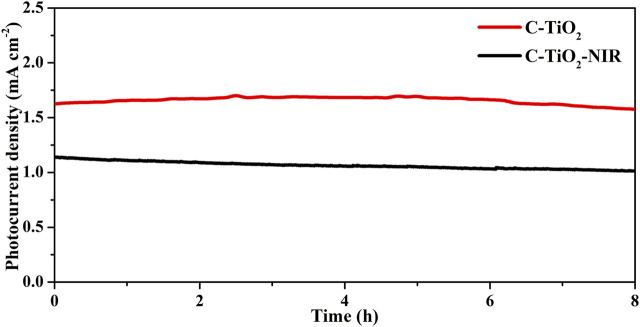
The stability of C-TiO_2_ and C-TiO_2_-NIR performed at 1.23 V vs. RHE for 8 h.

To confirm the important role that the photothermal effect plays in PEC water splitting, gas chromatography was used to examine the H_2_ and O_2_ evolution for the C-TiO_2_-NIR photoanodes. As shown in Figure 3D, the average H_2_ generation rate of C-TiO_2_-NIR reaches up to 25.84 μmol cm^−2^ h^−1^, while that of C-TiO_2_ is only 17.65 μmol cm^−2^ h^−1^. Additionally, a calculation of the Faraday efficiency yields 92.9% for C-TiO_2_-NIR, suggesting that the photocurrent density is derived from pure water splitting with the assistance of the photothermal effect. What’s more, the stability of C-TiO_2_ and C-TiO_2_-NIR was also investigated. As shown in Figure 4, the C-TiO_2_-NIR photoanode shows a stable operation for more than 8 h, only decreasing by ∼3% of its original photocurrent density value throughout the water splitting process, which is better than that of the C-TiO_2_ photoanode (decreasing by ∼11%).

**FIGURE 5:**
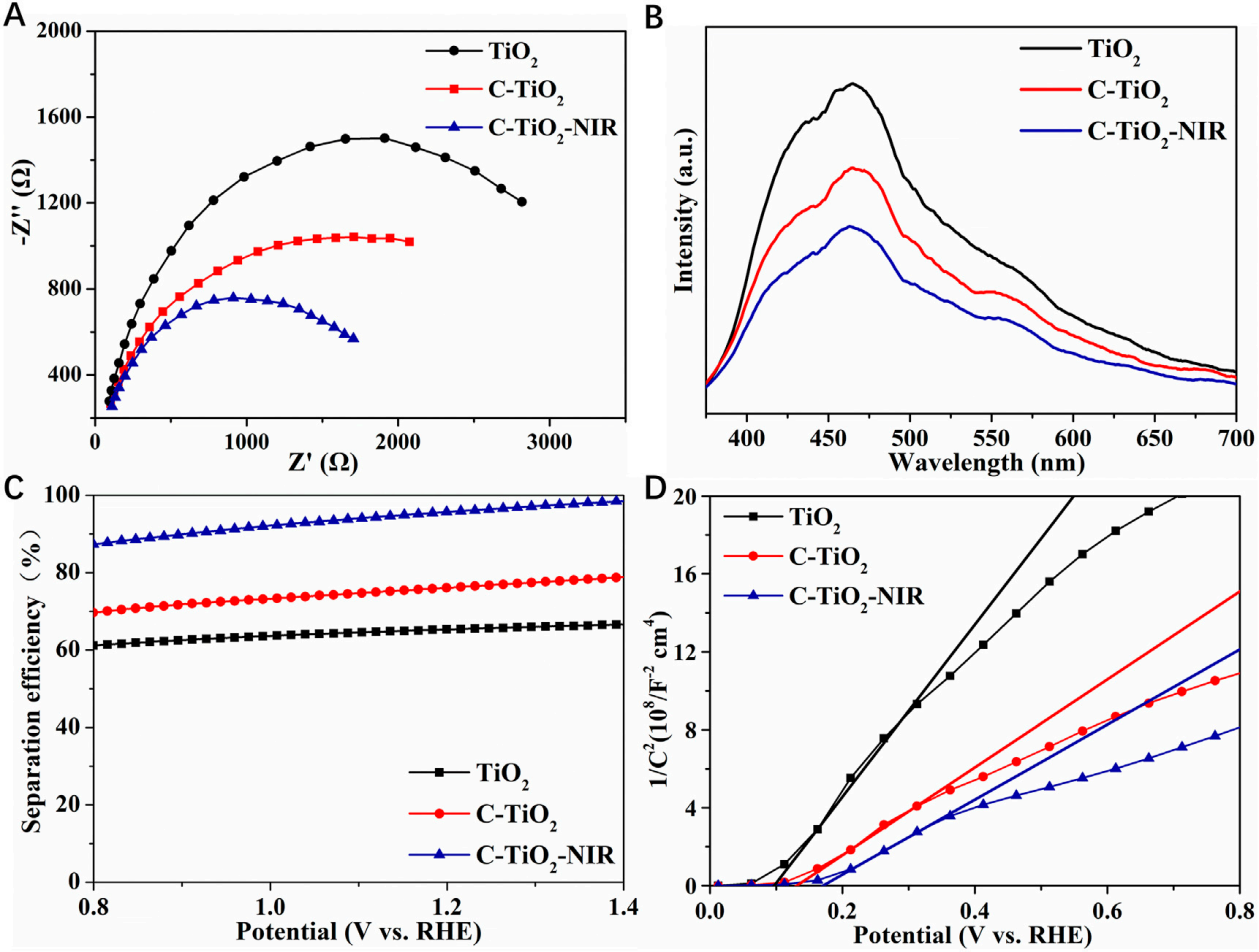
**(A)** EIS Nyquist plots, **(B)** PL spectra, **(C)** charge separation efficiency, **(D)** M–S plots of TiO_2_, C-TiO_2_, and C-TiO_2_-NIR.

To understand the mechanism of PEC performance enhancement induced by the photothermal effect, the charge transfer and water oxidation kinetics of the obtained photoanodes before and after NIR light irradiation were investigated thoroughly. The electron-hole pair recombination and charge generation kinetics of the photoanodes during the PEC water oxidation process can be analyzed by EIS using the results of impedance spectra to analyze electrochemical surface reactions. The charge transfer resistance of the photoanode surface is estimated from the small semicircle in the Nyquist diagram, and the smaller the radius, the more effective the separation of charges. As a result, the charge transfer resistance of C-TiO_2_ is lower than that of TiO_2_, suggesting facilitated interfacial charge transfer at the photoelectrode-electrolyte interface (Figure 5A). Additionally, C-TiO_2_-NIR shows the lowest charge transfer resistance, which indicates the enhanced interfacial charge transfer rate caused by the photothermal effect of CNPs. According to earlier research, the changed interfacial charge transfer could result from a lowering in the activation barrier for hole transfer at the TiO_2_/electrolyte interface as temperature rises. Furthermore, the thermal heating experiments (at around 43°C) of C-TiO_2_ photoanode were conducted, and compared with the NIR irradiated one. As shown in Supplementary Figure S2, the photocurrent density of C-TiO_2_ with thermal heating is slightly lower than that of C-TiO_2_ with the NIR irradiation, and the C-TiO_2_-NIR sample exhibits a relatively lower charge transfer resistance. The above results indicate that the C-TiO_2_ upon NIR irradiation can achieve better PEC performance due to the higher carrier densities in the bulk and faster charge transport rate at the surface induced by the photothermal effect.

To analyze the separation and recombination effects of photogenerated carriers on the photoanodes, the samples are explored by fluorescence spectroscopy (PL). The peak intensity of C-TiO_2_ and C-TiO_2_-NIR is much weaker than that of TiO_2_, which indicates that after CNPs loaded on the TiO_2_ surface and NIR light irradiation, the recombination of photogenerated electrons and holes is hindered and the charge separation efficiency is improved (Figure 5B). To quantify the effect of the photothermal properties of CNPs on the bulk charge separation of TiO_2_ photoanode, the efficiency of bulk charge separation (*η*_sep_) was investigated. As shown in Figure 5C, the separation efficiency of the TiO_2_ photoanode reaches about 66% at 1.23 V vs. RHE, while that of C-TiO_2_ and C-TiO_2_-NIR is 77% and 96% at 1.23 V vs. RHE, respectively. It reveals that the charge separation of photogenerated carriers is promoted because the electrons stored in CNPs are activated by NIR light and released rapidly. From this perspective, charge carriers can be more easily transported to the electrode surface/electrolyte interface due to the photothermal effect of CNPs, which contributes to water oxidation. The Mott-Schottky (M-S) plots were investigated to reveal the semiconductive properties of the obtained photoelectrode materials with and without NIR light irradiation (Figure 5D). It can be inferred from the positive slopes of the M-S plots for the photoanodes that all the samples are n-type semiconductors. The carrier densities C-TiO_2_ and C-TiO_2_-NIR are 1.30 × 10^20^ and 1.53 × 10^20^ cm^−3^, respectively. The greatly enhanced charge density might be associated with the improved electrical conductivity induced by the photothermal effect, which should facilitate charge separation (Yang et al., 2016).

**FIGURE 6:**
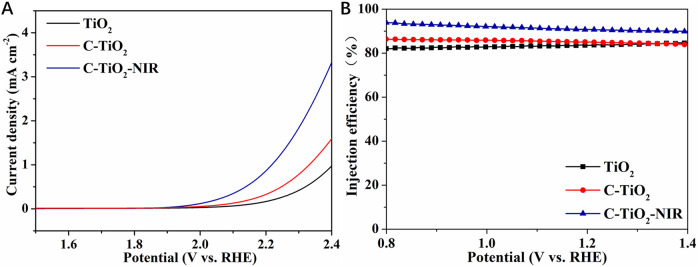
**(A)** The dark LSV measurements and **(B)** charge injection efficiency of TiO_2_, C-TiO_2_, and C-TiO_2_-NIR.

The LSV tests in dark conditions were performed to reveal the electrochemical water oxidation properties. As shown in Figure 6A, C-TiO_2_ photoanode shows a cathodic shift of the onset potential compared to the TiO_2_ anode, indicating the catalytic effect of CNPs. Importantly, the dark onset potential of C-TiO_2_-NIR is also cathodically shifted when irradiated by NIR light, suggesting the enhanced electrocatalytic water oxidation properties due to the photothermal effect of CNPs. In addition, the charge injection efficiency (*η*_inj_) was also calculated to investigate the water oxidation activities of the photoanodes, which shows that the *η*_*i*nj_ of the C-TiO_2_-NIR photoanode (91%) is higher than that of the TiO_2_ photoanode (84%) at 1.23 V vs. RHE (Figure 6B). Given that charge transport at the electrode/electrolyte interface is linked to the water oxidation rate, it can be inferred that the photothermal effect accelerates the water oxidation reaction. Combined with the *η*_sep_ results, it is demonstrated that the bulk electron-hole separation and the interface water oxidation rate of C-TiO_2_ and C-TiO_2_-NIR photoanodes are both increased by CNPs, which act as electron reservoirs as well as photothermal conversion materials. Consequently, the PEC performance of TiO_2_ photoanode is greatly improved.

**FIGURE 7:**
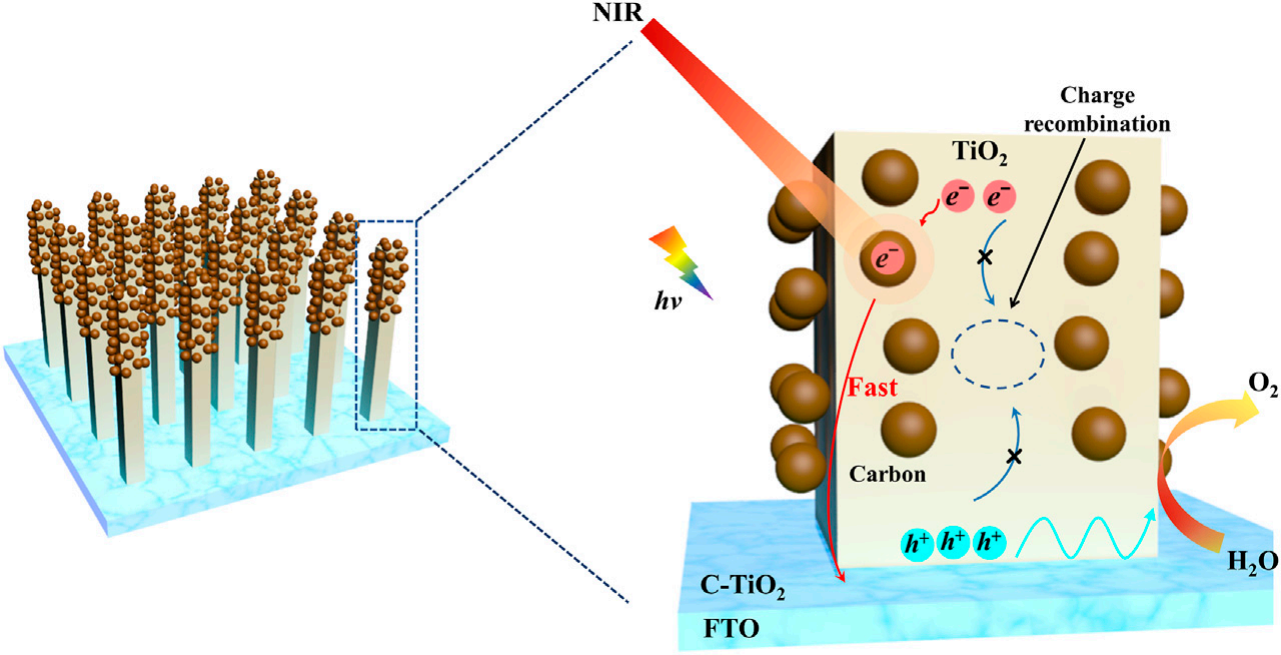
Schematic illustration of the photothermal-enhanced mechanism of PEC performance in the C-TiO_2_ photoanode system.

Based on the above results, the possible mechanisms for the improvement of the PEC activity of C-TiO_2_ photoanode and the photogenerated carrier transfer are shown in Figure 7. Acting as electron reservoirs, the CNPs can temporarily store the photogenerated electrons and efficiently separate the electron-hole pairs in the bulk of the C-TiO_2_ photoanode under solar light illumination. What’s more, when the photoanode is irradiated by NIR light, the local temperature on the surface of the photoanode is raised due to the photothermal effect of CNPs, which motivates the stored electrons to release and promotes the transport of bulk charge carriers and more holes to transfer to the photoelectrode/electrolyte interface, hence enhancing the water oxidation performance of C-TiO_2_ photoanode.

## Conclusion

In conclusion, we have prepared the C-TiO_2_ composite photoanode, and found that it leads to a remarkable enhancement in charge separation efficiency and water oxidation kinetics. The enhanced PEC performance of the C-TiO_2_ photoanode is attributed to the capture of photogenerated electrons by the CNPs as well as the photothermal effect. Irradiated by NIR light, the synergistic effect between the electron storage and the photothermal effect results in the fast bulk charge transport and surface oxidation kinetics of C-TiO_2_ photoanodes. Consequently, the photothermal-enhanced PEC performance of C-TiO_2_ reaches 1.62 mA cm^−2^ at 1.23 V_RHE_ under NIR light irradiation, with a high charge separation efficiency of 96%. The introduction of the photothermal effect proposed in this work provides a rational strategy to modify the PEC performance of photoelectrodes, which are expected to be widely developed for electrocatalysts, photocatalysts and other application fields.

## Data Availability

The original contributions presented in the study are included in the article/Supplementary Material, further inquiries can be directed to the corresponding author.
