# Supplementary material for: Enhancing the photoelectrochemical performance of TiO2 photoanode by employing carbon nanoparticles as electron reservoirs and photothermal materials
Source: Front Chem. 2024 Sep 24;12:1471340. doi: 10.3389/fchem.2024.1471340 (PMC11458437; doi:10.3389/fchem.2024.1471340)
Supplement: Supplementary file 1 [file DataSheet1.PDF]

Supplementary Material

Enhancing the Photoelectrochemical Performance of TiO_2 Photoanode by Employing Carbon Nanoparticles as Electron Reservoirs and Photothermal Materials

Yinchang Li, Yijie Huang, Puwen Guo, Jing Huang*

*Correspondence:

E-mail addresses: j.huang@hbnu.edu.cn

Supplementary Figures and Tables

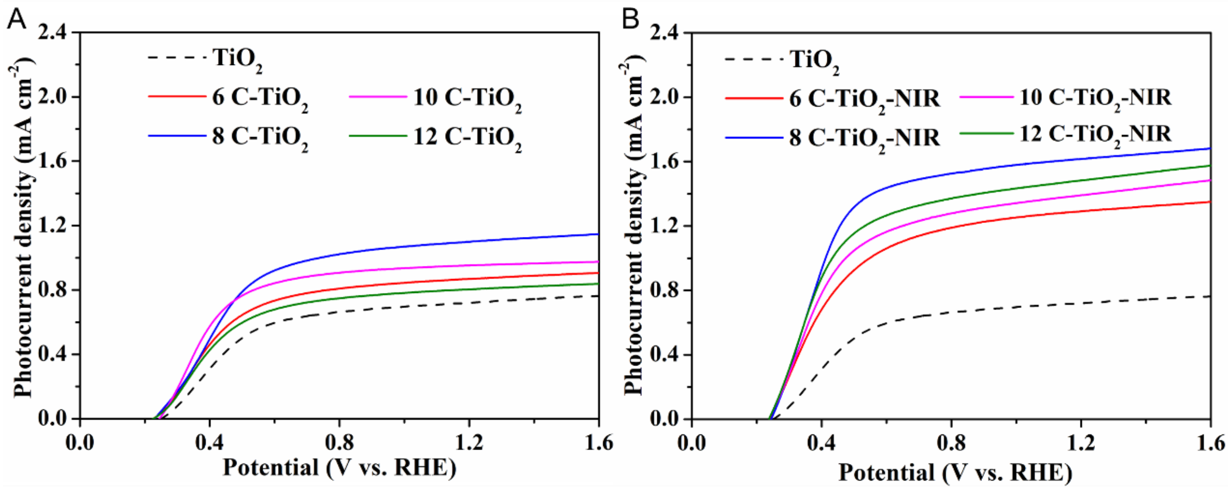

Supplementary Figure 1. LSV curves of TiO_2 and C-TiO_2 photoanodes prepared at 6 h, 8 h, 10 h, and 12 h: (A) with NIR light irradiation, (B) without NIR light irradiation.

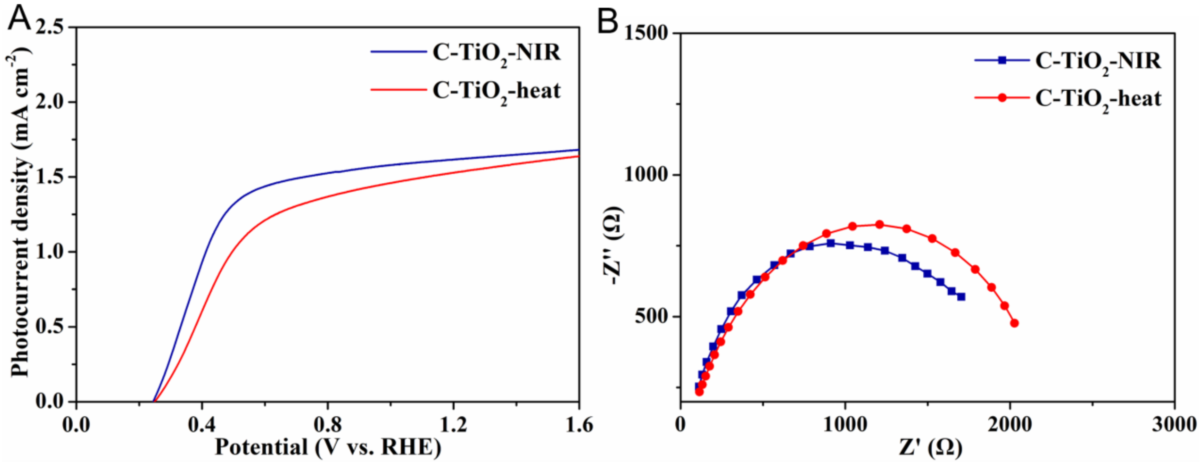

Supplementary Figure 2. The (A) LSV and (B) EIS curves of C-TiO_2 photoanodes tested under thermal heating and NIR light irradiation.

Samples	Test condition	V_{FB}（V）	N_d（cm^{-3}）	η_{sep}（%）
TiO_2	Stimulated solar light	0.10	6.65×10^{19}	66
C-TiO_2	Stimulated solar light	0.13	1.30×10^{20}	77
C-TiO_2	Stimulated solar light + NIR light	0.15	1.53×10^{20}	96

Supplementary Table 1. Characteristic parameters of TiO_2 and C-TiO_2 photoanodes.

Photoanodes	Photocurrent density	Test condition	Reference
Ag-TiO_2/blackphosphorus	1.63 mA cm^{-2} at 1.23 V_{RHE}	0.1 M Na_2SO_4	[1]
$NiCo_2O_4/TiO_2$	2.34 mA cm^{-2} at 1.23 V_{RHE}	1 M NaOH	[2]
BiOI/TiO_2 nanosheets	0.54 mA cm^{-2} at 1.23 V_{RHE}	0.5 M Na_2SO_4	[3]
Au-TiO_2 NS/TiO_2 NR	1.32 mA cm^{-2} at 1.23 V_{RHE}	1 M KOH	[4]
Co_3O_4/TiO_2	1.04 mA cm^{-2} at 1.23 V_{RHE}	1 M NaOH	[5]
TiO_2/MgAl-LDH	1.16 mA cm^{-2} at 1.23 V_{RHE}	1 M KOH	[6]
NiFe-MOF/TiO_2	0.77 mA cm^{-2} at 1.23 V_{RHE}	0.5 M Na_2SO_4	[7]
TiO_2/P-C_3N_4/Co_3O_4	1.58 mA cm^{-2} at 1.23 V_{RHE}	0.5 M Na_2SO_4	[8]
B-TiO_2/Au NPs/Au NRs	1.48 mA cm^{-2} at 1.23 V_{RHE}	0.5 M Na_2SO_4	[9]
TiO_2/CoP	1.53 mA cm^{-2} at 1.23 V_{RHE}	1 M KOH	[10]
O&H-Rutile TiO_2/Pd sample	1.50 mA cm^{-2} at 1.23 V_{RHE}	1 M NaOH	[11]
TiO_2-Ar	0.98 mA cm^{-2} at 1.23 V_{RHE}	1 M KOH	[12]
C-TiO_2-NIR	1.62 mA cm^{-2} at 1.23 V_{RHE}	1 M NaOH	This work

Supplementary Table 2. A comparison study between the C-TiO_2-NIR photoanode in this work and previously reported TiO_2-based photoanodes toward PEC water splitting.

References

[1] Liu Y, Jiang N, Lyu M, et al. TiO_2/black phosphorus heterojunction modified by Ag nanoparticles for efficient photoelectrochemical water splitting [J]. Materials Chemistry and Physics, 2023, 301: 127624.

[2] Huang J, Hu X, Wang J, et al. Unraveling photothermal-enhanced bulk charge transport and surface oxygen reactions in TiO_2 photoanodes for highly efficient photoelectrochemical water oxidation [J]. Chemical Engineering Journal, 2023, 462: 142246.

[3] Yang L, Wang R, Zhou N, et al. Construction of p-n heterostructured BiOI/TiO_2 nanosheets arrays for improved photoelectrochemical water splitting performance [J]. Applied Surface Science, 2022, 601: 154277.

[4] Cho S, Yim G, Park J T, et al. Surfactant-free one-pot synthesis of Au-TiO_2 core-shell nanostars by inter-cation redox reaction for photoelectrochemical water splitting [J]. Energy Conversion and Management, 2022, 252: 115038.

[5] Ding Q, Gou L, Wei D, et al. Metal-organic framework derived Co_3O_4/TiO_2 heterostructure nanoarrays for promote photoelectrochemical water splitting [J]. International Journal of Hydrogen Energy, 2021, 46(49): 24965-24976.

[6] Wang Y, Zhang Z, Wang S, et al. Integration of MgAl-layered double hydroxides into TiO_2 nanorods as photoanodes for enhanced photoelectrochemical water splitting [J]. Catalysis Communications, 2022, 164: 106434.

[7] Cui W, Bai H, Shang J, et al. Organic-inorganic hybrid-photoanode built from NiFe-MOF and TiO_2 for efficient PEC water splitting [J]. Electrochimica Acta, 2020, 349: 136383.

[8] Yu Z, Li Y, Qu J, et al. Enhanced photoelectrochemical water-splitting performance with a hierarchical heterostructure: Co_3O_4 nanodots anchored TiO_2@P-C_3N_4 core-shell nanorod arrays [J]. Chemical Engineering Journal, 2021, 404: 126458.

[9] Cao Z, Yin Y, Fu P, et al. Branched TiO_2 nanorod arrays decorated with Au nanostructure for plasmon-enhanced photoelectrochemical water splitting [J]. Journal of The Electrochemical Society, 2020, 167(2): 026509.

[10] Muhetaer M, Dong L, Wang Y, et al. CoP electrodeposited on TiO_2 nanorod arrays as photoanode for enhanced photoelectrochemical water splitting [J]. Journal of the American Ceramic Society, 2024: 1-10.

[11] Xu Y, Zhang C, Zhang L, et al. Pd-catalyzed instant hydrogenation of TiO_2 with enhanced photocatalytic performance [J]. Energy & Environmental Science, 2016, 9(7): 2410-2417.

[12] Huang H, Hou X, Xiao J, et al. Effect of annealing atmosphere on the performance of TiO_2 nanorod arrays in photoelectrochemical water splitting [J]. Catalysis Today, 2019, 330: 189-194.
